# Live Performance and Microbial Load Modulation of Broilers Fed a Direct-Fed Microbials (DFM) and Xylanase Combination

**DOI:** 10.3390/vetsci9030142

**Published:** 2022-03-18

**Authors:** Basheer Nusairat, Nasser Odetallah, Jeng-Jie Wang

**Affiliations:** 1 Department of Animal Production, College of Agriculture, Jordan University of Science and Technology, Irbid 22110, Jordan; 2 BioResource International, Inc., Durham, NC 27703, USA; nodetallah@briworldwide.com (N.O.); jwang@briworldwide.com (J.-J.W.)

**Keywords:** probiotic, xylanase, performance, lesion score, pathogen load, broiler

## Abstract

The animal industry, which focuses on producing protein for human consumption, is continuously seeking solutions that can enhance both animal performance and health at a low cost. Several feed additives are currently being used to improve the nutritive value of feed as well as replacing the subtherapeutic levels of antibiotic growth promoters (AGP). This study was designed to investigate the effect of a feed additive that is a blend of multi-strain *Bacillus* spp. probiotics and a xylanase in a 2 × 2 factorial dietary treatments design, testing two levels of the feed additive blend (0 and 100 g/MT) and two cereal grain types (corn and wheat) on live performance, gut lesions, environmental *Clostridium perfringens* load, and pathogen load in the digesta of broiler chickens (*E. tenella*, total aerobic count cells (APC), *E. coli*, and *C. perfringens*). Day-old chicks were randomly placed in 10 replicate pens per treatment with 52 birds per replicate and grown to 42 d of age. Data were analyzed by two-way ANOVA. At 42 d, birds fed EnzaPro were heavier (*p* < 0.0004) than unsupplemented birds. An improvement in FCR (*p* = 0.03) was observed from 1 to 42 d by approximately two points in both corn- and wheat-based diets supplemented with EnzaPro. In wheat-based diets, supplementing EnzaPro reduced (*p* < 0.0001) a 21 d lesion score of intestines with a further reduction (*p* < 0.02) at 42 d. EnzaPro reduced (*p* < 0.03) litter moisture by approximately 1% compared to non-supplemented EnzaPro in both corn- and wheat-based diets. Pathogen load in digesta (*C. perfringens*, *E. tenella*, APC, and *E. coli*) was reduced (*p* < 0.0002) when EnzaPro was supplemented in diets. It can be concluded that EnzaPro (a blend of DFM *Bacillus* spp (1 × 10^5^ CFU/g feed) and xylanase (10 XU/g feed)) may be used in both corn- and wheat-based diets to improve the performance and gut health of broilers.

## 1. Introduction

Feed additives are widely used in the animal industry, which is focused on producing protein for human consumption in mainly monogastric animals. The gastrointestinal tract of monogastric animals, mainly poultry, is either not producing a sufficient quantity of endogenous enzymes or lacking the enzymes necessary to break down the nutrients and antinutritional factors that are present in the feed [1]. Historically, exogenous enzymes were mainly used as feed additives to improve nutrient digestion in the feed. However, recently, the direction of feed additive utilization is shifting towards additional active substances other than enzymes. Such substances are expected to support the immune system of animals through different mechanisms of action, and thus, can be used as alternatives to the conventional antibiotic growth promoters (AGP).

Feed enzymes targeting improved nutrient digestibility have been shown to enhance live performance, while at the same time, allowing for the utilization of lower-quality feed ingredients, thus, giving flexibility to the formulation. Furthermore, a wide range of non-conventional byproduct ingredients can also be utilized when the proper combination of exogenous enzyme and substrate is established, eventually allowing for feed cost to be reduced as well as recycling byproducts to feasibly support sustainable agriculture.

One of the major limiting factors for feed ingredient incorporation in poultry rations is fiber content, mainly the non-starch polysaccharides (NSPs). NSPs are considered antinutritional substances and are indigestible by monogastric animals due to the lack of endogenous carbohydrases necessary for NSPs breakdown [2]. The NSPs exert their negative effect through entrapping nutrients (caging effect) and increasing digesta viscosity [3,4,5]. The NSPs are abundant in cereal grains such as corn and wheat which are the main energy sources (in the form of carbohydrates) and the most used feed ingredients in poultry diets worldwide. The presence of NSPs limits the potential of maximum energy digestibility in both corn- and wheat-soybean based diets. It has been reported that approximately an additional 450 kcal of digestible energy per kilogram of feed remains unutilized due to NSPs [6]. Several studies have shown that exogenous enzymes such as xylanase can release energy from the fiber fractions in the cell wall of corn, wheat and other ingredients, resulting in fewer nutrients available for opportunistic microorganisms in the lower gut and, subsequently, improving broiler performance and return on investment [7,8,9,10,11,12,13,14].

An additional benefit of using xylanases in monogastric animal diets is that NSPs are degraded by xylanase into smaller oligosaccharides units of xylose and arabinose as the major products; these molecules could serve as prebiotic compounds that can be utilized by beneficial bacteria in lower gut [15]. Ding et al. [16] reported that these xylooligosaccharides stimulate the growth of Gram-positive bacteria such as *Bifidobacteria* and *Lactobacilli*. Therefore, xylanase can provide both nutritional and gut health benefits to the animal by improving digestibility and providing prebiotics as a source of nutrition for beneficial bacteria.

Recently, nutritionists started recognizing the importance of both host and microbiota health. The relationship between gut microbiota and animal health is mainly influenced by nutrition and environment. Specifically, gut-associated immunity represents a major component of the bird’s overall health and is eventually shaped by the microflora, colonizing the gut during the first few weeks post-hatch as acquired through early-life feeding and environment. The immune system in poultry is partially developed at hatch [17], while the gastrointestinal tract is sterile [18] and eventually mature by 3 weeks of age; therefore, it is important to provide the optimum environment and feed to promote the proper development of both systems.

The concept of early probiotics feeding in poultry started in the early 1970s when Nurmi and Rantala [19] published in *Nature* the successful elimination of *Salmonella enteritidis* in newly hatched chicks by feeding a suspension of the gut contents collected from healthy adult chickens, which supported healthy microflora development in the newly hatched chicks and competitive exclusion of pathogenic microorganisms. The supplementation of probiotics has shown promise in the development of the microflora, which plays a major role in maintaining intestinal health by competitive exclusion, reduction in pathogenic microbes colonization, and decrease in the energy expenditure of the immune system, thus, improving the productivity of chicken, eventually leading to the benefits reflected in the improved broiler performance [20,21,22,23].

It is hypothesized that the combination of xylanase and probiotic strains could provide beneficial effects on the nutrition of both the host and microorganisms, as well as promoting beneficial microflora in a relationship that can be described as a symbiotic relationship, simultaneously providing a fiber-degrading enzyme, prebiotic, and probiotic benefits to the host and beneficial microorganisms, as well as developing an overall healthy/host-preferred microbiota balance. Therefore, this study was performed to investigate the effect of xylanase and its probiotic effect on live performance, gut lesions, environmental *Clostridium perfringens* load, and pathogen load in the digesta of broiler chickens raised on used litter and fed either corn- or wheat-based diets to 42 days of age.

## 2. Materials and Methods

Animal care practices conformed to the Guide for the Care and Use of Agricultural Animals in Agricultural Research and Teaching [24].

### 2.1. Experimental Design

A total of 2080 Ross 708 mixed sex 1 d-old broilers were obtained from a commercial hatchery and randomly placed in floor pens (305 cm × 137 cm) with 10 replicate pens per treatment, each containing 52 chicks and raised to 42 days of age under typical US broiler production conditions in a completely randomized design with 2 × 2 treatment factorial arrangement. The chicks were reared on used litter, spiked with *C. perfringens* (10^7^ CFU/bird) at day 1 and Eimeria species at day 7 (*E. acervulina*: 10^4^ CFU/bird, and *E. tenella*: 10^6^ CFU/bird), and at day 10 with *E. maxima* (10^3^ CFU/bird). The birds were given ad libitum access to feed and water. The lighting program included 23 h of light for the first week at minimum intensity of 3-foot candles (fc) dimmed to 1 fc for the remainder of the trial.

### 2.2. Experimental Diets

A total of four factorially arranged dietary treatments were designed to evaluate the effect of a feed additive—that is, a blend of xylanase and multi-strain *Bacillus* spp. probiotics (EnzaPro, BioResource International Inc., Durham, NC, USA)—at 2 levels (0 and 100 g/MT), and 2 cereal grain types (corn and wheat). Table 1 illustrates the dietary formulation and nutrient composition for starter, grower, and finisher phases for both corn- and wheat-based treatments. EnzaPro was added at the level of 100 g/MT, which provided 10 XU (xylanase unit) of endo-β-1,4-xylanase, and the probiotics provided 1 × 10^5^ CFU of multi-strain *Bacillus* spp. per gram of feed. Both xylanase activity and *Bacillus* spp. enumeration were confirmed by analyzing the feed samples. The diets were formulated to either meet or exceed the nutrient requirements of broilers [25] according to strain producer nutrition specification recommendation [26]. The birds were fed mash starter (days 1 to 21), grower (days 22 to 35), and finisher diets (day 36 to 42).

### 2.3. Data Collection

#### 2.3.1. Live Performance

The birds and feed were weighed at placement and at 21, 35, and 42 days for live performance measurements. Mortality was recorded as it occurred. The measurements were used for determining body weight (BW); body weight gain (BWG); feed intake (FI); feed conversion ratio (FCR), adjusted for mortality as listed in the equation below; BW coefficient of variation as an indicator of flock uniformity (CV—flock uniformity); and percent mortality. Flock uniformity was based on the coefficients of variation using individually measured BWs and by assessing how each BW deviated from the mean BW of each pen.

FCR, adjusted for mortality, = [Total FI for a period]/{[Total days of birds alive at the end of the period] + [Total days of birds culled/dead]}/{[Total pen weight gained in the period*] + [Total weight of culled/dead birds in this period]}/{[Total days of birds alive at the end of the period] + [Total days of birds culled/dead]}.

#### 2.3.2. Apparent Metabolizable Energy Digestibility

On days 19 and 40, 4 birds per pen (2 males and 2 females) were randomly selected and moved to raised-wire cages. The birds were fasted for 6 h followed by feeding of the respective diets until days 21 and 42, respectively. Feed consumption was measured per cage. All excreta were collected during the feeding period, as well as during the 42 h after feed removal (post-feeding portion). Excreta samples were pooled, dried, processed and analyzed for dry matter, gross energy, and nitrogen. The feed consumed was also analyzed for dry matter, gross energy, and nitrogen. The following calculations were used to determine apparent metabolizable energy (AME) and nitrogen corrected (AMEn):

AMEn = [FI × GEfeed) − (DMfecal × GEfecal) − (NR × 8.73)]/FC

where FI = feed intake; GEfeed = gross energy of feed; DMfecal = fecal dry matter; GEfecal = gross energy of feces; NR = nitrogen retention, where NR = (FC × feed nitrogen)-(DMfecal × fecal nitrogen).

#### 2.3.3. Intestinal Lesion Score

At 21 and 42 d, 2 birds from each sex per pen were randomly selected and tested for intestinal lesions in the small and large intestines as an indicator of coccidiosis caused by *Eimeria* spp. The lesions were scored by trained personnel based on the presence and/or severity of any intestinal lesions using a scoring range from 0 (no lesions found) to 4 (actual bleeding observed) as illustrated by Johnson and Reid [27]. Scores were based on lesions in the entire intestines.

#### 2.3.4. *E. tenella* Enumeration, Pathogen Load in the Digesta, and Salmonella Incidence

Enumeration of *E. tenella* was performed on cecal content, results expressed as log_10_ oocyst per bird. The pathogen load for total aerobic count (APC), *Clostridium perfringens*, and *E. coli* were enumerated using the digesta from small intestine collected on 21 and 42 days of age; from each pen, 2 birds from each sex were sampled. The results were expressed as log_10_ CFU/g, while *Salmonella* incidence was expressed as a percentage (%).

#### 2.3.5. Litter Moisture

The moisture of the litter was measured on days 0, 21, and 42 of age. Samples were taken from 3 sites of each pen and pooled together; then, samples were oven-dried following the procedure set out in the AOAC [28] and litter moisture expressed as a percentage (%).

#### 2.3.6. Environmental Pathogen Load

The counts of *Clostridium perfringens* (*C. perfringens*) in the litter were enumerated as an indicator of environmental pathogen load. *C. perfringens* colony-forming units (CFUs) per gram of pen litter were measured prior to placement, and at 21, and 42 days of age per FDA BAM, Ch 16 [29]. Briefly, 25 g of litter was homogenized in 225 mL of peptone diluent (0.1% peptone); then, 10-fold dilutions of each sample were prepared up to 10^9^. One milliliter of each dilution was placed on tryptose sulfitecycloserine agar plates and incubated under anaerobic conditions at approximately 35 °C for approximately 24 h. The plates were then removed from the incubator and the total viable *C. perfringens* colonies were counted using dilution plates with approximately 20–200 CFUs. The samples were analyzed in quadruplicate.

### 2.4. Statistical Methods

The data were analyzed as 2 × 2 factorial in a completely randomized design with 10 replicate pens per dietary treatment and 20 replicates per main effect (cereal grain and EnzaPro). The general linear model of SAS (Statistical Analysis System, 2017) was employed. The means were separated by LSMEANS. An arc–sin transformation was applied to the percentage values before testing for differences. The superscripts were determined based on PDIFF values. The experimental unit for live performance parameters, digestibility, pathogen load in ceca, digesta, and litter was the pen. Means were considered significantly different at a set *p* value of ≤0.05.

## 3. Results

**Figure 1:**
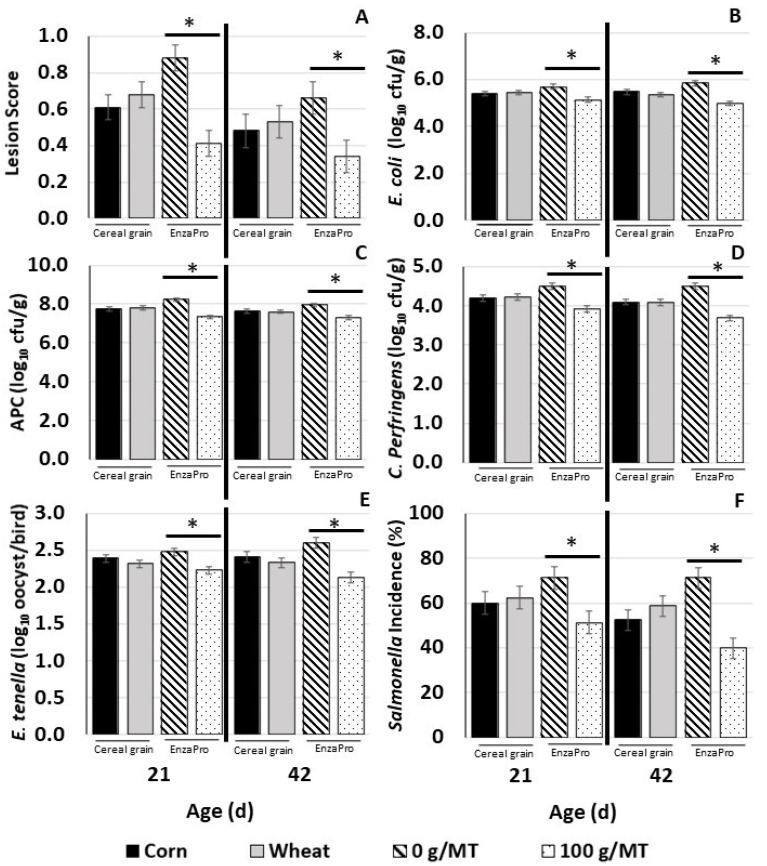
Least-square means for intestinal coccidia lesion scores and pathogen load measured at 21 and 42 d of age for the main effects of cereal grain (corn and wheat) and EnzaPro (0 and 100 g/MT). (**A**) Lesion scores in broiler intestines at 21 and 42 d; (**B**) *E. coli* log_10_ cfu/g counts in digesta; (**C**) aerobic plate count log_10_ cfu/g counts in digesta; (**D**) *C. perfringens* log_10_ cfu/g counts in digesta; (**E**) *E. tenella* log_10_ oocyst per bird counts in cecal contents; (**F**) *Salmonella* incidence % in digesta. * within each plot indicates a significant difference within each main effect at *p* ≤ 0.05.

Table 2, Table 3 and Table 4 and Figure 1 report the least-square means of the main effects of cereal grain and EnzaPro; there were no interactions between the main effects, indicating that enzyme supplementation was independent from cereal grain type. Only the main effect outcomes are reported and discussed.

There were no significant differences in the main effect of cereal grain in any of the measured performance parameters, energy digestibility, intestinal lesion score, pathogen load in digesta and environment, *Salmonella* incidence, and litter moisture; therefore, the results will focus on EnzaPro’s main effects.

### 3.1. Live Performance and Apparent Metabolizable Energy Digestibility

The live performance results are reported in Table 2 (FI, BW, BWG, FCR, CV of BW, and mortality). EnzaPro’s supplementation effect was significantly observed on body weight and body weight gain. The influence on body weight and body weight gain was observed first at 21 d and increasing at 42 d, yielding an approximate 2% improvement in body weight at 42 d due to EnzaPro supplementation. However, differences in body weight gain did not reach significance in the last week of finisher phase (36–42 d). The mortality and BW coefficient of variation (an indicator of flock uniformity) were not affected by cereal grain type or EnzaPro supplementation.

The results for the main effect of cereal grain and EnzaPro on apparent metabolizable energy (AME) and apparent metabolizable energy corrected for nitrogen (AMEn), calculated at both 21 and 42 d, are presented in Table 3. Supplementing EnzaPro increased (*p* < 0.05) AME and AMEn at both 21 and 42 d (Table 3) by approximately 50 kcal/kg, which translates to an approximate 1.7% and 1.4% increase in AME and AMEn, respectively, regardless of age.

### 3.2. Intestinal Lesion Score, Pathogen Load in the Digesta, *E. tenella* Enumeration, and Salmonella Incidence

Figure 1 summarizes the results for lesion scores measured in the small and large intestines at 21 and 42 d, pathogen load in digesta, and *E. tenella* oocyst count in ceca of birds at 21 and 42 d. Since there was no interaction between cereal grain and EnzaPro, only the main-effect results are presented.

EnzaPro supplementation reduced (*p* < 0.05) lesion score at both 21 d and 42 d by approximately 50% and 30%, respectively. Additionally, EnzaPro supplementation reduced (*p* < 0.05) counts of *E. coli*, APC, and *C. perfringens* by an average of 1 log_10_ cfu/g; incidences of *Salmonella* in digesta samples by about 30%; and *E. tenella* oocyst counts by approximately 0.5 log_10_ oocyst per bird in cecal samples.

### 3.3. Litter Moisture and Environmental Pathogen Load

The main effects for cereal grain and EnzaPro supplementation on litter moisture and *C. perfringens* counts are presented in Table 4. EnzaPro supplementation reduced (*p* < 0.05) the moisture of litter at 42 d by 3% compared to the 0 g/MT treatment.

For the counts of *C. perfringens* in the litter, there was no effect of cereal grain on litter *C. perfringens* counts, while EnzaPro supplementation tended (*p* < 0.10) to decrease counts at 21 d by 41%. The counts were numerically lower at 42 d.

## 4. Discussion

The absence of interactions between the main effects indicate that enzyme supplementation was independent from cereal grain type. The measured performance parameters were not affected by cereal grain type; similar results were previously reported when feed intake was compared by Tang et al. between corn, wheat, barley, and sorghum as the sole source of cereal grain in a dietary treatment [30]; they reported that feed intake was higher in birds consuming corn from 0 to 21 d but was comparable between birds fed corn and wheat from 0 to 42 d. Body weight and body weight gain from 0 to 42 d were also comparable between birds fed corn- and wheat-based rations [30]. In the current trial, wheat dietary treatments were formulated with both wheat (15%) and corn (reduced by approximately 18% compared to the pure corn dietary treatments) as cereal grain sources, which could explain the lack of significant differences between corn and wheat. However, this also confirms that live performance will not be adversely affected by using wheat at 15% in broiler rations. Farahat et al. [31] compared corn-based to wheat-/barley-based broiler rations and showed that feed intake and body weight gain were not affected by cereal type from 0 to 42 d. Several other researchers showed that cereal grains did not have a significant effect on performance—even though, in some of these studies, a significant effect on early-life performance parameters was noticed, but these differences disappeared by 42 d of age [32,33]. On the other hand, EnzaPro supplementation improved body weight and body weight gain without significantly affecting feed intake, which supports the finding that body weight improvements were not driven by feed intake, but rather by supplementation. These findings are consistent with the previously published effect of EnzaPro supplementation on the performance of broilers [18,34]. These studies also reflected on the improved (*p* < 0.05) FCR from 1 to 42 d, which is consistent with the current trial, with two points of FCR improvement due to EnzaPro supplementation. Several researchers reported improved broiler body weight gain and FCR due to either probiotic supplementation alone or when combined with xylanase under diverse pathogen challenge models [35,36]. Furthermore, Singh et al. [37] reported that xylanase supplementation promoted beneficial bacteria in the gut of broilers, which led to improved performance. This supports the hypothesis that a synergistic effect exists between xylanase and probiotics when combined.

Overall, mortality was less than 2%, which is within the expected mortality rate under commercial settings. This mortality rate indicates that the mild microbial challenge employed did not result in the high rate of secondary infection that would cause increased mortality, but rather was reflected in a depressed performance.

Flock uniformity was higher than 10% at 21 d but improved afterwards, achieving a rate that was less than 10%. However, there was no dietary treatment effect on flock uniformity. The improved flock uniformity has been previously discussed by Nusairat and Wang [13] as it is related to mixed-sex pens and differences in nutrient partitioning and distribution between males and females [38].

The AME and AMEn measured at both 21 and 42 d were not affected by cereal grain, but were improved by EnzaPro supplementation. It was expected that wheat-based diets would result in reduced energy digestibility, since wheat contains more-soluble NSPs than corn, which may increase digesta viscosity [39], thus influencing digestibility more than corn. However, these outcomes were not observed, which was probably be due to the use of only 15% wheat in the wheat dietary treatments. Tang et al. [30] did not observe any differences in AME between corn- and wheat-based diets at 21 or 41 d when investigated alongside sorghum and barely, even when each grain was used as the sole source in the diets. Furthermore, several researchers have shown the improvement not only for AME, but also other nutrients by xylanase supplementation [40,41,42,43]. Kouzounis et al. [44] investigated wheat-based diets and showed improved nutrient digestibility and arabinoxylan fermentability in broilers. It is well-established that NSPs, although considered antinutritional factors, are also nutrient-rich when combined with the compatible enzyme that functions to release these nutrients; furthermore, this lowers viscosity, thus leading to improved digestion and absorption efficiency.

The type of cereal grain had no effect on intestinal lesion score, pathogen load in the digesta, *E. tenella* enumeration, or *Salmonella* incidence parameters measured at 21 and 42 d. Previous research supports these findings, where Farahat et al. [31] reported that there was no effect on total bacteria and the coliforms of cecal contents of broilers fed either corn- or wheat/barley-based diets. However, Lactobacilli counts were higher in corn-fed broilers. In addition, Paraskeuas and Mountzouris [45] did not observe significant difference in *E. coli* counts between corn- and wheat-based broiler diets. On the other hand, EnzaPro supplementation reduced these parameters at both 21 and 42 d. This confirms previous results by Nusairat et al. [34], who reported the beneficial effect of xylanase and probiotic combination on reducing lesion scores, intestinal pathogen load, and *Salmonella* incidence in broilers at 21 and 42 d. Several studies have investigated different direct-fed microbials (DFM) that possess gut-health-enhancing capabilities in both chicken and turkey; *Lactobacilli* supplementation was shown to reduce cecal coliform counts in both broilers and turkey [46,47] and reduce both *Salmonella enteritidis* and *Clostridium perfringens* in chicken [48]. Furthermore, xylanase supplementation alone has been shown to reduce counts of *E. coli* and increase counts of *Lactobacillus* spp. in the ceca of 35 d-old broilers [15]; therefore, the reduced microbial load and lesion scores could be due to the combined effect of both the xylanase and probiotic multi-strain present in EnzaPro. Reducing the pathogen counts in the digesta and ceca indicates that fewer pathogens are also shed into the environment, thus controlling the spread of infection among the flock and subsequent flocks, since the industry in the US relies on recycled bedding for poultry production.

Litter moisture was not affected by cereal grain, while EnzaPro reduced litter moisture. In general, litter moisture content increased with the increasing age of birds, confirming that, as the flock gets closer to marketing, managing moisture in litter becomes crucial to avoid welfare violations such as footpad lesions, as well as increased ammonia in broiler houses [49]. Furthermore, litter moisture could be used as an indicator of flock health when environmental management is applied successfully; less wet litter indicates fewer wet excreta, which relates to the proper digestion process; thus, fewer nutrients are excreted into the environment. Therefore, there may be a reduced concentration of opportunistic pathogens in litter [50], which may play a key role in the emergence of human food-borne pathogens if not controlled. Therefore, litter moisture can be reduced by supplementing 100 g/MT of EnzaPro in broiler rations.

The effect of EnzaPro on *C. perfringens* was not strong enough to yield a significant difference. *C. perfringens* can be found naturally in the gastrointestinal tract of poultry and in the environment. It is associated with necrotic enteritis, and it cannot be easily controlled. It can be classified as an opportunistic pathogen that would take advantage of damaged epithelial cell wall to exert its effect. Promoting healthy gut microflora, as well as reducing litter moisture, could aid in minimizing favorable conditions for *C. perfringens* to express its virulency [51,52,53,54].

## 5. Conclusions

The supplementation of a combination of direct-fed microbials and xylanase at a level of 1 × 10^5^ CFU/g feed and 10 XU/g feed, respectively, has proven its beneficial effects when fed to broilers from day 1 to marketing and exposed to mild disease challenge, utilizing both *Eimeria* and *C. perfringens*. This beneficial effect was due to the simultaneous introduction, regardless of the cereal grain used. Xylanase’s effect extends beyond simply reducing the viscosity of digesta to facilitate nutrient digestion and absorption; rather, it is hypothesized to have created a prebiotic effect that indirectly promoted a healthy gut microflora which, in combination with the muti-strain probiotic used, provided an optimum “starter culture” early in the birds’ lives that aided in shaping a beneficial microflora to support the growth and health of broiler by day 21. Therefore, it can be concluded that the supplementation of 100 g/MT of EnzaPro can support body weight gain and reduce the pathogen load in broilers raised to 42 d under mild disease conditions.

## Figures and Tables

**Table 1:** Composition and nutrient content of experimental diets.

Ingredient (%)	Starter (1–21 d)		Grower (22–35 d)		Finisher (36–42 d)	
	Corn	Wheat	Corn	Wheat	Corn	Wheat
Corn	58.16	46.32	64.50	52.67	68.94	57.14
Wheat	-	15.00	-	15.00	-	15.00
Soybean meal 48%	36.30	32.62	29.49	25.78	23.34	19.63
Poultry meal	0.71	1.15	1.73	2.17	3.83	4.26
Poultry fat	0.05	0.05	0.05	0.05	0.05	0.05
DL-Methionine	0.25	0.23	0.19	0.18	0.08	0.06
Salt	0.49	0.49	0.45	0.44	0.39	0.38
Lysine	0.00	0.09	0.04	0.14	0.03	0.13
Limestone	1.60	1.59	1.32	1.32	1.22	1.21
Dicalcium phosphate	1.89	1.91	1.68	1.70	1.57	1.59
Vitamin and mineral premix ^1^	0.50	0.50	0.50	0.50	0.50	0.50
Sand filler ^2^	0.05	0.05	0.05	0.05	0.05	0.05
Calculated nutrients (%)						
Metabolizable energy (kcal/kg)	2900	2900	3000	3000	3100	3100
Crude protein	22	22	20	20	19	19
Crude fat	1.44	1.39	1.87	1.84	1.81	2.12
Crude fiber	2.62	2.68	2.59	2.64	2.55	2.60
Ash	7.84	8.81	7.46	7.55	8.77	7.31
Calcium	1.05	1.05	0.9	0.9	0.85	0.85
Available phosphate	0.50	0.50	0.45	0.45	0.42	0.42
Sodium	0.22	0.22	0.2	0.2	0.18	0.18
Dig Lysine	1.28	1.28	1.15	1.15	1.02	1.02
Dig Methionine + cysteine	0.947	0.947	0.851	0.851	0.755	0.755
Analyzed nutrients (%)						
Crude protein	22.8	21.3	18.9	18.9	17.8	17.6
Crude fat	1.70	1.63	1.57	1.44	1.67	1.77
Crude fiber	3.01	2.84	2.87	2.99	2.72	2.58
Ash	6.55	6.58	6.10	6.54	7.95	7.35

^1^ Vitamin and trace mineral premix supplied the following per kg of diet: 5512 IU vitamin A, 1852 IU vitamin D3, 11 IU vitamin E, 0.06 mg vitamin B12, 0.23 mg biotin, 1.87 mg menadione (K3), 0.44 mg thiamine, 3.75 mg riboflavin, 5.95 mg d-pantothenic acid, 1.32 mg vitamin B6, 34.17 mg niacin and 0.22 mg folic acid, for mineral supplied the following per kg of diet: manganese: 120 mg, zinc: 120 mg, iron: 80 mg, copper: 10 mg, iodine, 2.5 mg, cobalt, 1 mg. ^2^ Sand filler was used to allow for EnzaPro incorporation in 100 g/MT EnzaPro treatments by replacing 0.01% (100 g/MT) of sand at each phase. EnzaPro provided 10 XU (xylanase unit) of endo-β-1,4-xylanase, and the probiotics provided 1 × 10^5^ CFU of multi-strain *Bacillus* spp. per gram of feed.

**Table 2:** Least-square means for feed intake (FI), body weight (BW), body weight gain (BWG), feed conversion ratio (FCR) corrected for mortality, mortality, and BW coefficient of variation for broilers raised to 42 d.

	Dietary Treatments					*p*-Value		
	Cereal Grain		EnzaPro (g/MT)		SEM ^1^	*Cereal grain*	*EnzaPro*	*Cereal grain x EnzaPro*
Age Period, d	Corn	Wheat	0	100				
FI, g/bird								
1–21	1042	1044	1039	1047	4.11	0.834	0.347	0.883
22–35	2466	2484	2471	2479	10.3	0.388	0.702	0.333
36–42	1383	1360	1363	1379	18.4	0.552	0.673	0.468
1–42	4890	4888	4873	4905	14.9	0.934	0.298	0.787
BW, g/bird								
Day 0	47.7	47.5	47.8	47.4	0.162	0.698	0.241	0.435
Day 21	838	837	832 ^b^	843 ^a^	2.85	0.843	0.0479	0.894
Day 35	2137	2133	2117 ^B^	2153 ^A^	5.67	0.718	0.0008	0.916
Day 42	2815	2813	2788 ^B^	2841 ^A^	7.86	0.841	0.0004	0.908
BWG, g/bird								
1–21	791	790	784 ^b^	796 ^a^	2.83	0.859	0.0395	0.856
22–35	1299	1296	1285 ^b^	1310 ^a^	5.75	0.822	0.0298	0.872
36–42	679	680	671	687	8.53	0.961	0.363	0.975
1–42	2768	2765	2740 ^B^	2793 ^A^	7.86	0.848	0.0004	0.923
FCR, g:g								
1–21	1.32	1.32	1.32	1.31	0.0024	0.354	0.0368	0.756
22–35	1.80	1.82	1.83	1.80	0.0079	0.198	0.101	0.436
36–42	1.87	1.84	1.87	1.85	0.0268	0.601	0.771	0.547
1–42	1.72	1.72	1.73 ^a^	1.71 ^b^	0.0044	0.642	0.0282	0.822
Mortality, %								
1–21	1.44	1.54	1.35	1.63	0.253	0.855	0.583	0.583
22–35	0.240	0.350	0.335	0.255	0.0865	0.541	0.656	0.656
36–42	0.210	0.105	0.210	0.105	0.0885	0.567	0.567	0.567
1–42	1.87	1.98	1.87	1.98	0.283	0.859	0.860	0.601
BW Coefficient of variation, %								
21	13.7	14.1	13.9	13.9	0.144	0.255	0.945	0.858
35	8.95	8.85	8.88	8.93	0.0885	0.585	0.788	0.564
42	9.30	9.12	9.48	9.04	0.0966	0.630	0.0251	0.934

^a,b^ means in a row within each main effect that lack common superscript differ significantly (*p* ≤ 0.05). ^A,B^ means in a row within each main effect that lack common superscript differ significantly (*p* ≤ 0.01). ^1^ SEM: standard error of the mean for *n* = 40 pens.

**Table 3:** Least-square means for apparent metabolizable energy (AME) and apparent metabolizable energy corrected for nitrogen (AMEn) for broilers raised to 42 d.

	Dietary Treatments					*p*-Value		
	Cereal Grain		EnzaPro (g/MT)		SEM ^1^	*Cereal Grain*	*EnzaPro*	*Cereal Grain x EnzaPro*
	Corn	Wheat	0	100				
AME (kcal/kg)								
Day 21	2923	2922	2898 ^B^	2947 ^A^	5.03	0.818	0.0001	0.951
Day 42	2892	2890	2866 ^B^	2916 ^A^	4.72	0.801	0.0001	0.937
AMEn (kcal/kg)								
Day 21	3121	3119	3096 ^B^	3143 ^A^	5.14	0.764	0.0001	0.989
Day 42	3091	3089	3067 ^B^	3113 ^A^	4.70	0.679	0.0001	0.883

^A,B^ means in a row within each main effect that lack common superscript differ significantly (*p* ≤ 0.01). ^1^ SEM: standard error of the mean for *n* = 40 pens.

**Table 4:** Least-square means for litter moisture and environmental pathogen load indicators.

	Dietary Treatments					*p*-Value		
	Cereal Grain		EnzaPro (g/MT)		SEM ^1^	*Cereal Grain*	*EnzaPro*	*Cereal Grain x EnzaPro*
	Corn	Wheat	0	100				
Litter moisture, %								
Day 0	18.5	18.8	18.7	18.6	0.132	0.237	0.501	0.885
Day 21	22.9	22.9	22.9	23.0	0.129	0.916	0.896	0.697
Day 42	26.9	27.3	27.5 ^a^	26.6 ^b^	0.211	0.311	0.0307	0.889
Litter *Clostridia perfringens,* log_10_ CFU/g								
Day 0	4.36	4.18	4.19	4.35	0.114	0.424	0.496	0.967
Day 21	4.17	4.04	4.31	3.90	0.107	0.542	0.0601	0.319
Day 42	3.56	4.08	4.16	3.88	0.0939	0.544	0.147	0.811

^a,b^ means in a row within each main effect that lack common superscript differ significantly (*p* ≤ 0.05). ^1^ SEM: standard error of the mean for *n* = 40 pens.

## Data Availability

The raw data have not been published or stored anywhere else; however, they are available upon request from Basheer Nusairat.
